# Supplementary material for: Nuclear deformability increases PARPi sensitivity in BRCA1-deficient cells by increasing microtubule-dependent DNA break mobility
Source: Nat Commun. 2025 Jun 17;16:5326. doi: 10.1038/s41467-025-60756-8 (PMC12174318; doi:10.1038/s41467-025-60756-8)
Supplement: Supplementary file 1 — Supplementary Information [file 41467_2025_60756_MOESM1_ESM.pdf]

Supplementary Information

Supplementary Figure 1

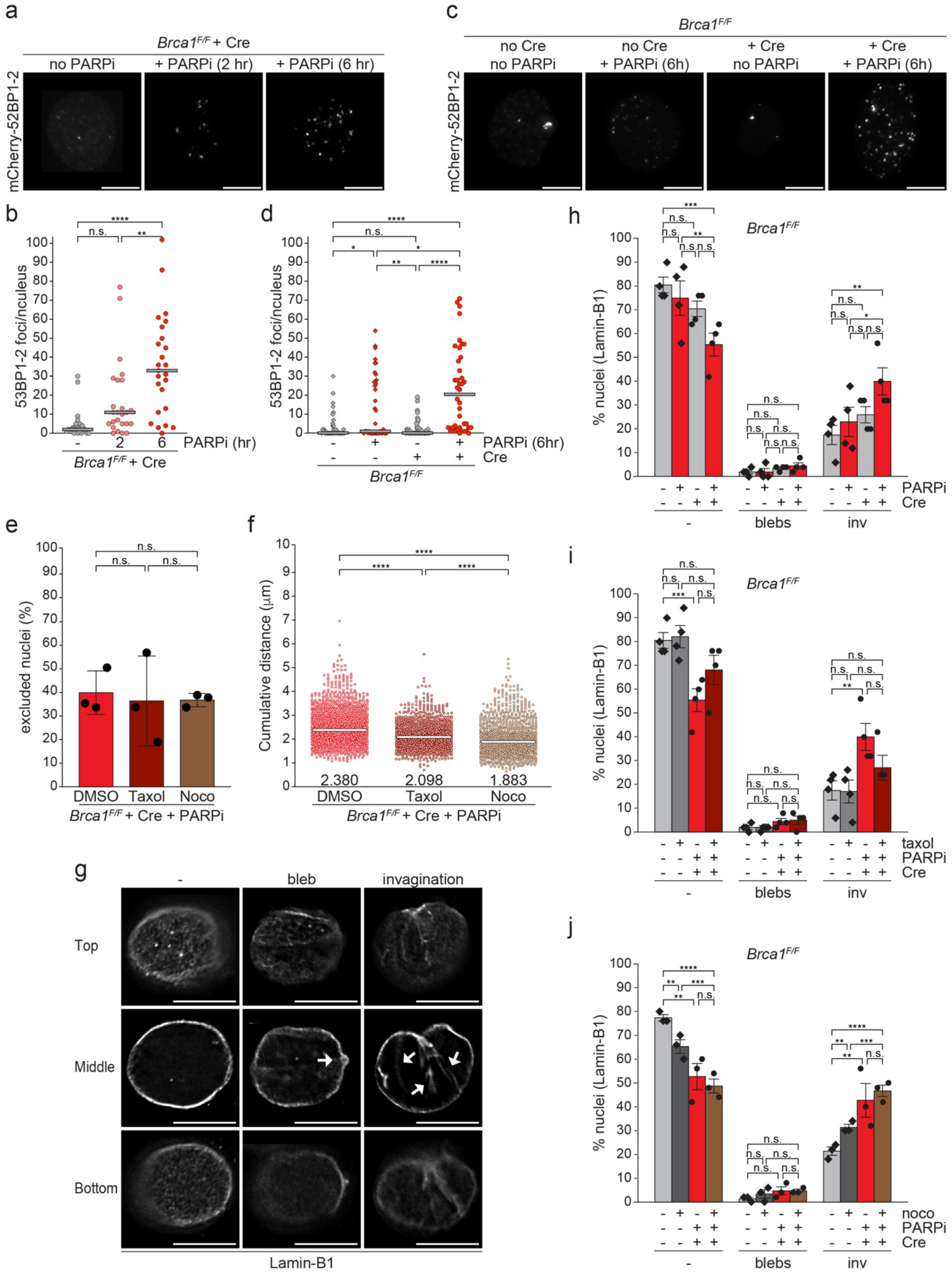

Supplementary Figure 1: DSBs mobility and nuclear deformations in BRCA1-deficient cells treated with PARPi

(a) Representative images of mCherry-BP1-2 foci in *Brca1*$^{F/F}$ MEFs 72 h after *Brca1* deletion and 2 or 6 h after PARPi addition or in the absence of any further treatment. (b) Quantification of mCherry-BP1-2 foci per nucleus in the MEFs treated as in (a) in one representative experiment, with median (n=25). (c) Representative images of mCherry-BP1-2 foci in *Brca1*$^{F/F}$ MEFs without any treatment or 72 h after BRCA1 deletion and/or 6 h PARPi addition. (d) Quantification of mCherry-BP1-2 foci per nucleus in the MEFs treated as in (c) in one representative experiment, with median (n=40). (e) Quantification of nuclei excluded from further analysis due to large-scale deformations as detected by tracing the mobility of the mCherry-53BP1 foci 72 h after *Brca1* deletion with Hit&Run Cre and 6 h after treatment with PARPi and DMSO, taxol (1 h) or nocodazole (2 h). Data from at least 15 nuclei for condition for each experiment over n=3 independent experiment. Total nuclei analyzed: 58 for DMSO, 47 for taxol, and 44 for nocodazole. (f) Cumulative distance (CD) of mCherry-BP1-2 foci in the MEFs as described in (e), with median (total foci analyzed: 1618 for DMSO, 1056 for taxol, and 1085 for nocodazole from 35, 30, 28 nuclei, respectively). (g) Representative images of nuclei with no deformations (-), blebs, or invaginations by Immunofluorescence (IF) with anti- Lamin-B1 antibodies in MEFs. Cells were fixed before immunostaining. Arrows indicate blebs/invaginations. (h) Quantification of nuclei with no deformations (-), blebs (blebs), or invagination (inv) as shown in (g) in *Brca1*$^{F/F}$ MEFs without any treatment or 72 h after *Brca1*$^{F/F}$ deletion and/or 6 h PARPi addition. Data are from 200 nuclei from n=4 independent experiments (50 cells/experiment) with with mean +/- SEM. (i-j) Quantification of nuclear deformations as described in (g-i) in the same *Brca1*$^{F/F}$ MEFs without any treatment or 72 h after *Brca1* deletion and 6 h PARPi addition in the absence or presence of taxol for 1 hours (i) or nocodazole for 2 hours (j). Data are from 200 nuclei from n=4 independent experiments or 150 nuclei from n=3 independent experiments (50 cells/experiment), respectively, with mean +/- SEM.

The experiments in (h-j) were performed together.Statistical analysis by ordinary One-way ANOVA for multiple comparisons for (b, d, e), Mann-Whitney test t-test for (f), or 2way Anova Tukey's multiple comparisons test (h) or 2way Anova Šídák's multiple comparisons test for the indicated pairs (i-j): (****) p<0.0001, (***) p<0.001, (**) p<0.01, (*) p<0.05, (ns) not significant.

Source data are provided as a Source Data file.

Supplementary Figure 2

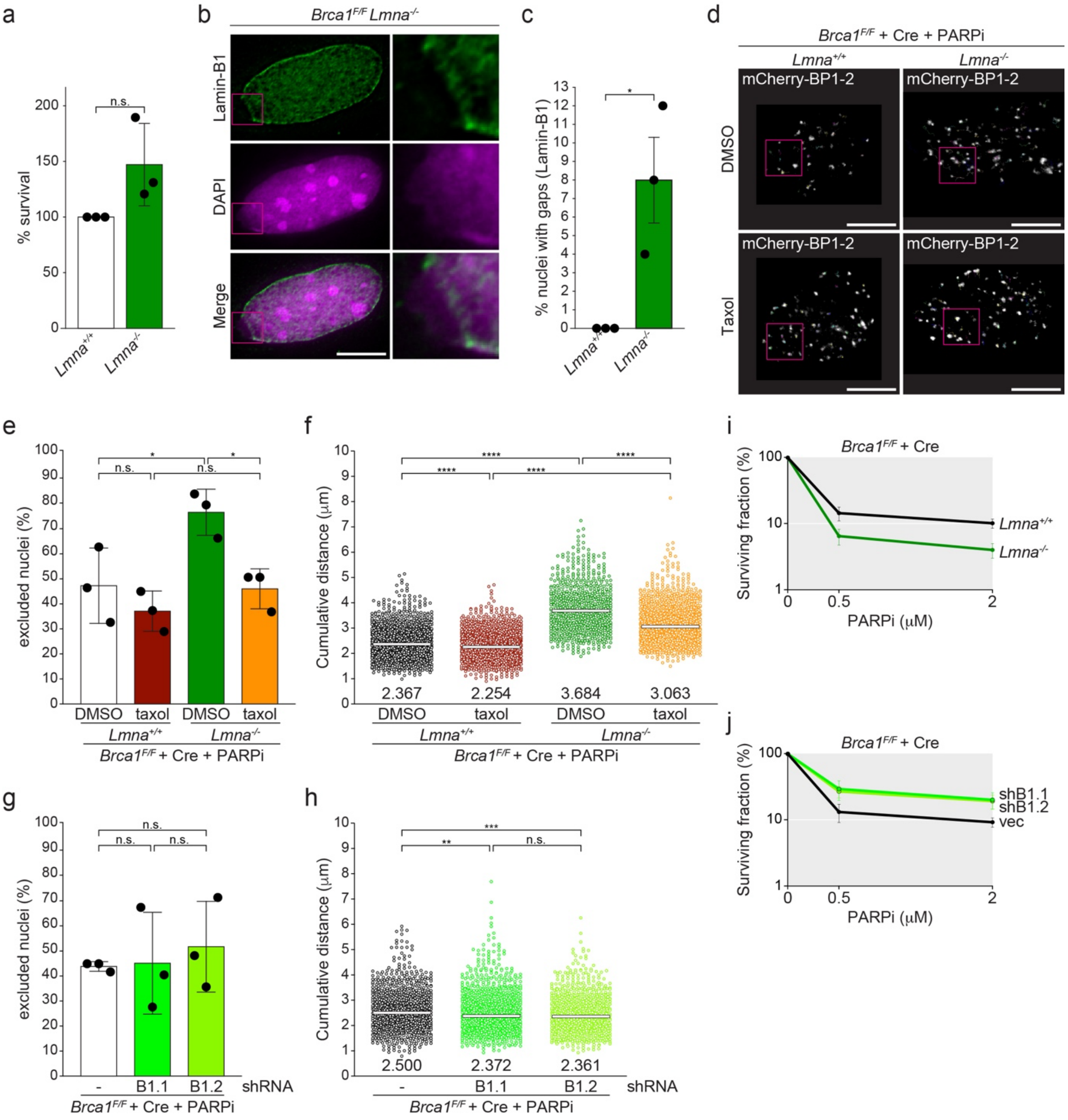

Supplementary Figure 2: Lamin-A but not Lamin-B1 suppresses DSBs mobility in BRCA1-deficient cells treated with PARPi

(a) Quantification of survival of *Brca1*$^{F/F}$ *Lmna*$^{+/+}$ or *Brca1*$^{F/F}$ *Lmna*$^{-/-}$ MEFs without any treatment. Colonies were stained with Methylene blue and their number in the different conditions was normalized over the number of colonies recovered for the *Brca1*$^{F/F}$ *Lmna*$^{+/+}$ control cell line. Data from n=3 independent experiments, with mean and SD.
One sample t test and Wilcoxon. (b) Representative images of IF with anti- Lamin-B1 antibodies (green) of *Brca1*$^{F/F}$ *Lmna*$^{+/+}$ or *Brca1*$^{F/F}$ *Lmna*$^{-/-}$ MEFs. Cells were fixed before immunostaining. DAPI is shown in magenta. Square indicates a gap (= substantial interruption in the peripheral staining pattern of Lamin-B1). (c) Quantification of Lamin-B1-negative gaps in MEFs deleted of *Lmna* as shown in (a) for n=3 independent experiments. For each experiment, 50 cells were analyzed for condition as in Fig. 2d,e. (d) Examples of 10 min traces of mCherry-BP1-2 foci in *Brca1*$^{F/F}$ *Lmna*$^{+/+}$ or *Brca1*$^{F/F}$ *Lmna*$^{-/-}$ MEFs s 72 h after *Brca1* deletion, 6 h after PARPi addition in the absence or presence of taxol as shown

enlarged in Fig. 2g. Scale bars, 10 μm. (e) Quantification of discarded nuclei as detected by analyzing the mobility of the mCherry-53BP1 foci 72 h after *Brca1* deletion with Hit&Run Cre, 6 h after treatment with PARPi and/or taxol treatment for 1 h, as described in (d). Data from at least 15 nuclei for condition for each experiment over n=3 independent experiment. Total nuclei analyzed: 76 for *Lmna*$^{+/+}$, 58 for *Lmna*$^{+/+}$+ taxol, 89 for *Lmna*$^{-/-}$ and 68 for *Lmna*$^{-/-}$ + taxol. (f) Cumulative distance (CD) of mCherry-BP1-2 foci in the MEFs as described in (d) and (e), with median (total foci analyzed over n=3 independent experiment: 1442 for *Lmna*$^{+/+}$, 1487 for *Lmna*$^{+/+}$+ taxol, 711 for *Lmna*$^{-/-}$ and 1374 for *Lmna*$^{-/-}$ + taxol from 42, 37, 21, 37 nuclei, respectively). (g) Quantification of discarded nuclei in *Brca1*$^{F/F}$ *Lmna*$^{+/+}$ transduced with the empty vector control (vec) or two shRNAs against (mRNA) *LmnB1* (B1), as detected by analyzing the mobility of the mCherry-53BP1 foci 72 h after *Brca1* deletion with Hit&Run Cre and 6 h after treatment with PARPi. Total nuclei analyzed over n=3 independent experiments: 53 for vec, 67 for shB1.1, 55 for shB1.2. (h) CD of mCherry-BP1-2 foci in the MEFs as described in (f), with median (total foci analyzed: 998 for vec, 1250 for shB1.1, and 959 for shB1.2, from 30, 36, and 27 nuclei respectively). (i) Quantification of survival of *Brca1*$^{F/F}$ *Lmna*$^{+/+}$ or *Brca1*$^{F/F}$ *Lmna*$^{-/-}$ MEFs after cre-mediated deletion of *Brca1* and treated with the indicated concentrations of PARPi as shown in Fig.3e but normalized over no PARPi treatment. Data from n=3 independent experiments, with mean and SEM. (j) Quantification of survival of *Brca1*$^{F/F}$ *Lmna*$^{+/+}$ MEFs transduced with vec, shB1.1, or shB1.2 after Cre-mediated deletion of *Brca1* and treatment with the indicated concentration of PARPi as shown in Fig.3f but normalized over no PARPi treatment. Data from n=3 independent experiments, with mean and SEM. Statistical analysis by unpaired t-test for (a) and (c), ordinary One-way ANOVA for multiple comparisons for (e) and (g), and Mann-Whitney test t-test for (f) and (h).
Source data are provided as a Source Data file.

Supplementary Figure 3

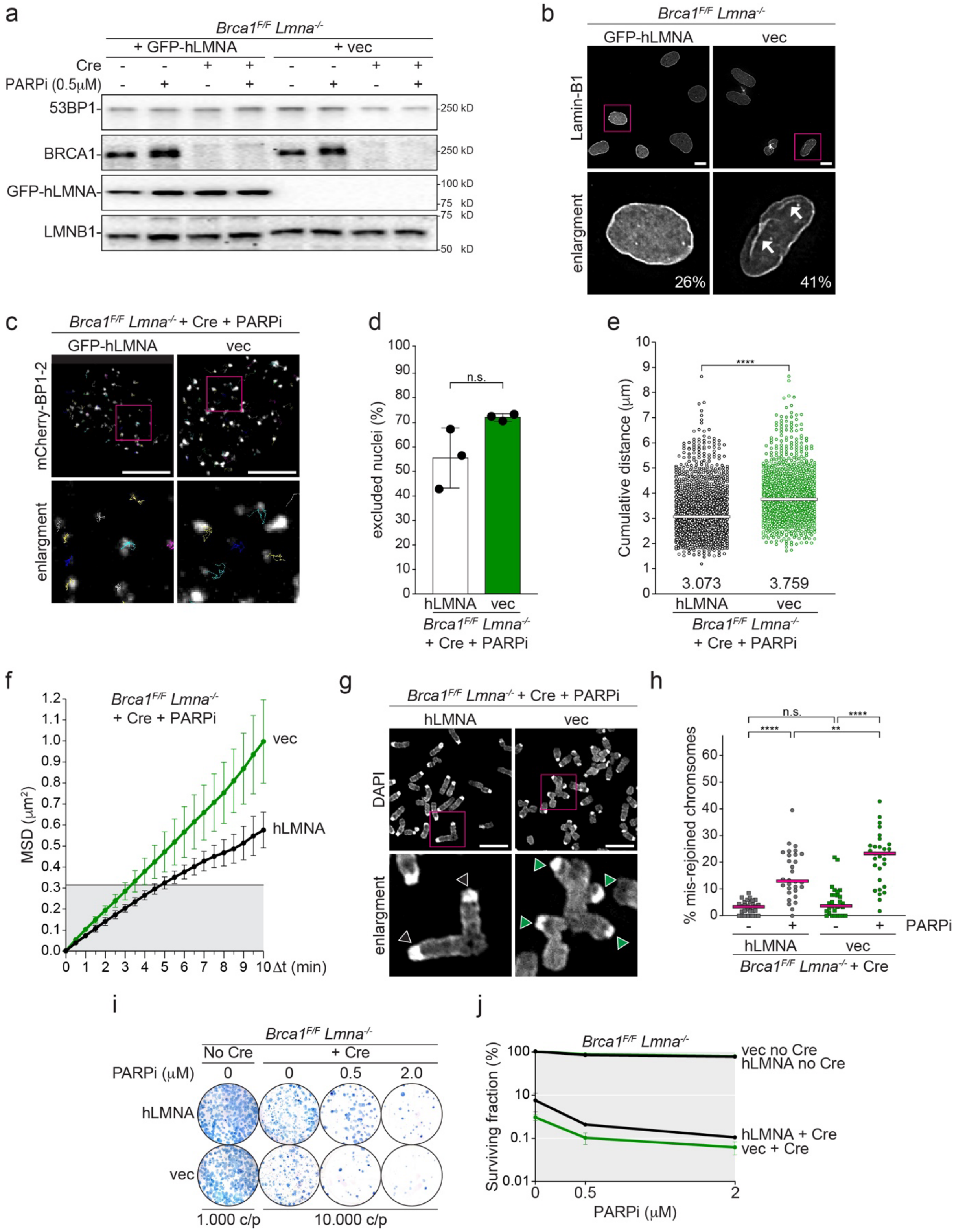

Supplementary Figure 3: Ectopic overexpression of hLMNA partially complement *Lmna* deletion in BRCA1-deficient cells treated with PARPi

(a) Immunoblot for 53BP1, BRCA1, GFP-hLMNA and LMNB1 in sgP53-immortalized *Brca1*$^{F/F}$ *Lmna*$^{-/-}$ MEFs complemented with the empty vector control (vec) or GFP-hLamin-A (hLMNA), without any treatment or 72 h after *Brca1* deletion with Hit&Run Cre and/or

addition of PARPi, as indicated. (b) IF for Lamin-B1 of representative nuclei of Brca1F/F Lmna-/- MEFs complemented with the empty vector control (vec) or GFP-hLMNA, with quantification of NE invaginations in one representative experiment. GFP-hLMNA n=68; vec n=66. (c) Examples of 10 min traces of mCherry-BP1-2 foci in *Brca1*$^{F/F}$ *Lmna*$^{-/-}$ MEFs complemented with vec or hLMNA 72 h after *Brca1* deletion and 6 h treatment with PARPi. (d) Discarded nuclei of MEFs treated as described in (c). Data from at least 15 nuclei for condition for each experiment over n=3 independent experiment (total nuclei analyzed: 85 for hLMNA and 95 for vec). (e) Cumulative distance with median, and (f) MSD with SD of mCherry-BP1-2 foci in the same MEFs as described in (c) and (d) for 39 nuclei for hLMNA and 27 nuclei for vec (total foci: 1530 for hLMNA and 1174 for vec). In (f), the line indicates the final MSD reached in *Brca1*$^{F/F}$ *Lmna*$^{+/+}$ MEFs, as shown in Fig.2h. (g) Representative mis-rejoined chromosomes in *Brca1*$^{F/F}$ *Lmna*$^{-/-}$ MEFs complemented with vec or hLMNA 96h after *Brca1* deletion with Hit&Run Cre and PARPi treatment for 24h. DNA is visualized with DAPI. Arrowheads indicate mis-rejoining events. (h) Quantification of the percentage of mis-rejoined chromosomes per metaphases as shown in (g). Each dot represents a metaphase. Bars represent the median for n=3 independent experiments, with 10 metaphases each (30 metaphases in total). (i) Representative survival assay of *Brca1*$^{F/F}$ *Lmna*$^{-/-}$ MEFs complemented with vec or hLMNA treated with or without hit&Run Cre and/or the indicated concentration of PARPi. Cells were treated with PARPi for 24h before wash. (j) Quantification of survival as shown in (i). Colonies were stained with Methylene blue and their number in the different conditions was normalized over the number of colonies recovered in the untreated plates. Data represents the average and SEM from n=3 independent experiments. Statistical analysis by unpaired t-test for (d), Mann-Whitney test t-test for (e) and ordinary One-way ANOVA for multiple comparisons for (h). All scale bars, 10 μm.
Source data are provided as a Source Data file.

Supplementary Figure 4

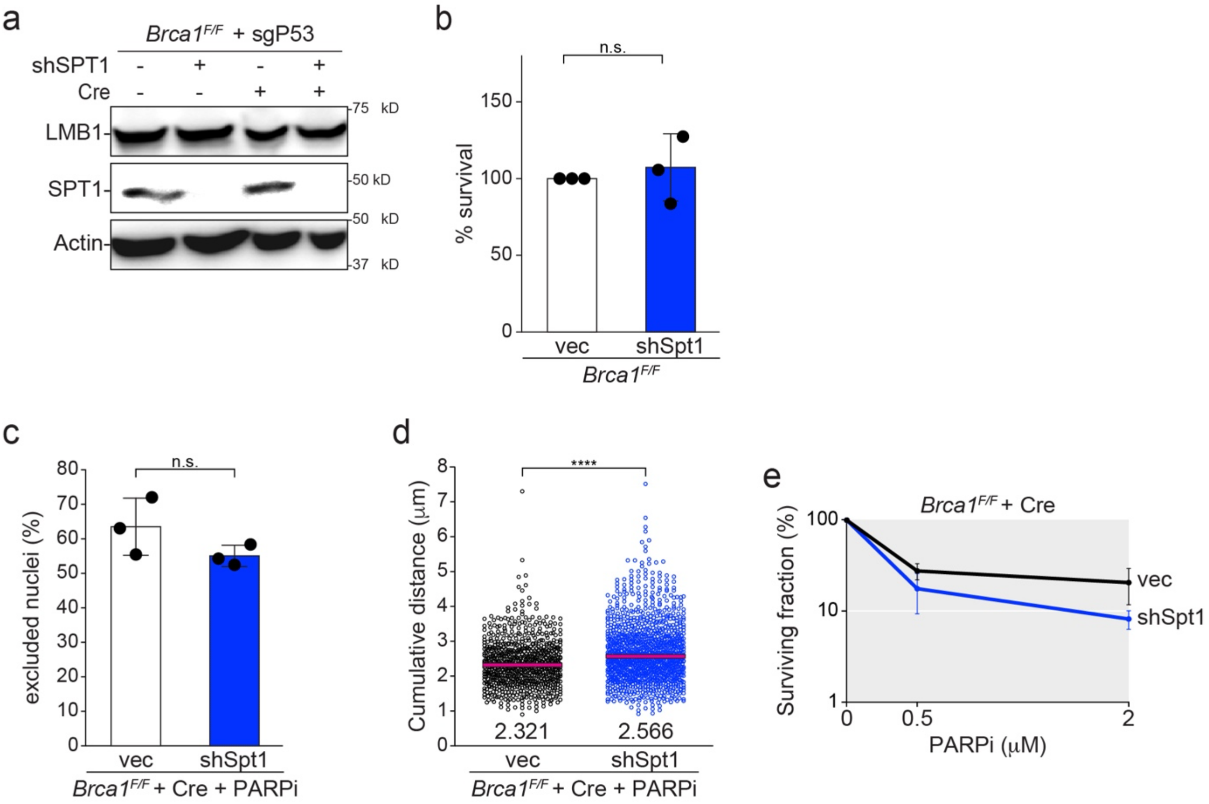

Supplementary Figure 4: SPT1 depletion increase DSBs mobility

(a) Immunoblot for LMNB1, SPT1 and actin as loading control in sgP53-immortalized *Brca1*$^{F/F}$ MEFs transduced with the empty vector control (vec) or an shRNA against (mRNA) *Spt1* (shSpt1) before or 72 hr after Hit&Run Cre-mediated deletion of *Brca1*. (b) Quantification of survival of *Brca1*$^{F/F}$ MEFs transduced with the empty vector control (vec) or shSPT1 without any treatment. Colonies were stained with Methylene blue and their number in the different conditions was normalized over the number of colonies recovered for the vec control. Data from n=3 independent experiments, with mean and SD. (c) Quantification of discarded nuclei in *Brca1*$^{F/F}$ MEFs transduced with or without shSpt1 as detected by analyzing the mobility of the mCherry-53BP1 foci 72 h after BRCA1 deletion with Hit&Run Cre and 6 h after treatment with PARPi as described in Fig. 4. Data from at least 15 nuclei for condition for each experiment over n=3 independent experiment (total nuclei analyzed: 65 for vec and 70 for shSpt1). (d) CD of mCherry-BP1-2 foci in the included MEFs as described in (c). Graph shows CD of 763 (vec) or 1201 (shSpt1) foci from 24 and 32 cells, respectively, with median. (e) Quantification of survival of *Brca1*$^{F/F}$ MEFs transduced with vec or shSpt1 after Cre-mediated deletion of *Brca1* and treatment with the indicated concentration of PARPi as shown in Fig.4i but normalized over no PARPi treatment. Data from n=3 independent experiments, with mean and SEM. Statistical analysis by One sample t test and Wilcoxon (b), un-paired t-test and (c), Mann-Whitney test t-test d). Source data are provided as a Source Data file.

Supplementary Figure 5

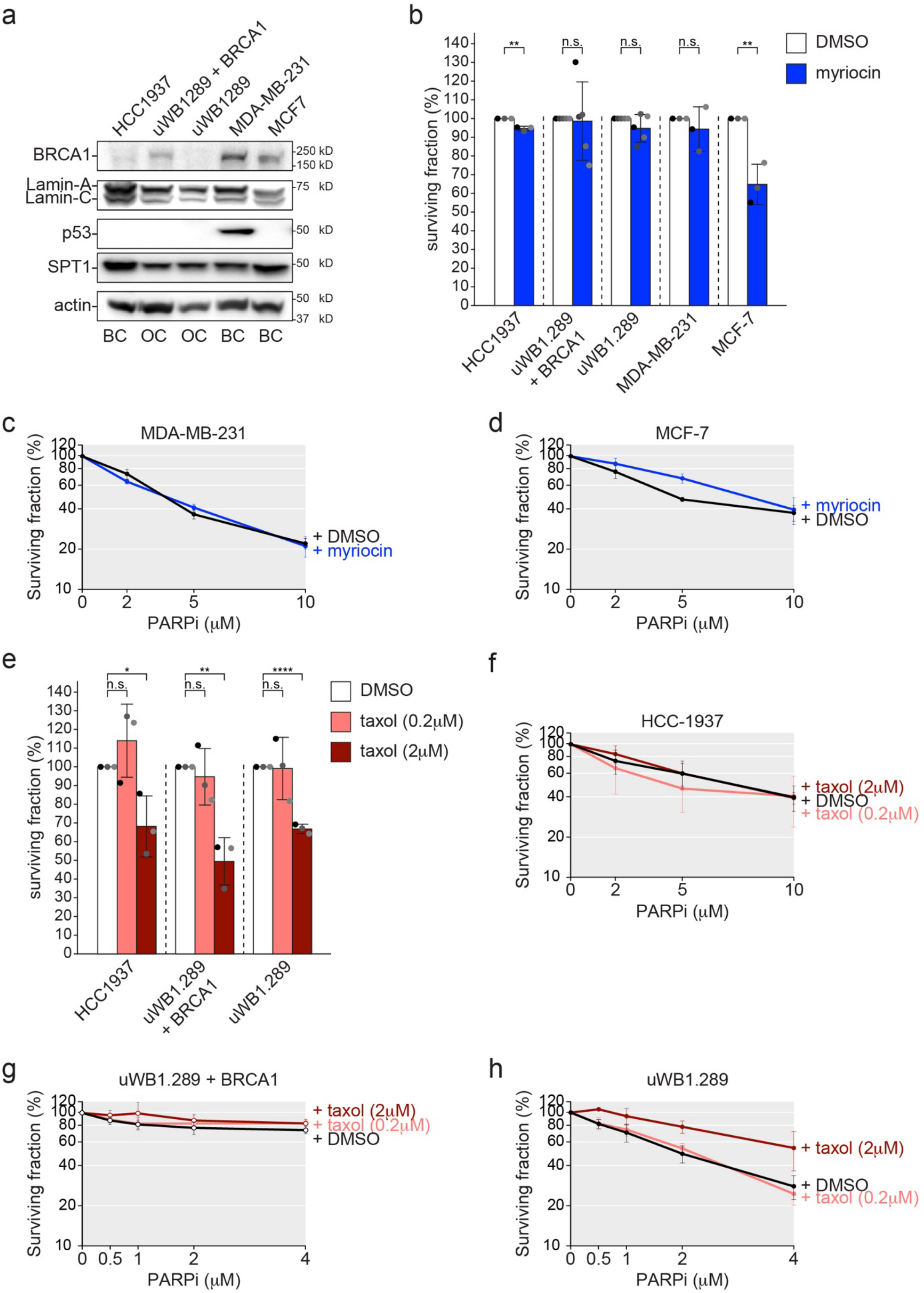

Supplementary Figure 5: SPT inhibition increase PARPi-sensitivity of BRCA1-deficient cancer cells

(a) Immunoblot for BRCA1, LMNA/C, p53, SPT1 and actin in the indicated breast (BC) or ovarian (OV) cancer cell lines. (b) Survival assay for the indicated breast (BC) or ovarian (OV) cancer cell lines after exposure for 24 h to myrocin. After removal of the drugs, cells were let grow for a week before harvest and counting. Quantifications of growth is normalized over the untreated conditions. Data from n=3/5 independent experiments, as indicated, with mean and SD. (c-d) Quantification of survival of MDA-MB-231 (c) or MCF7 (d) breast cancer cell line after exposure for 24 h to myrocin and/or 72 h exposure to PARPi at the indicated concentrations. After removal of the drugs, cells were let grow for a week before harvest. Cells were counted after trypsinization. Quantifications of growth is normalized over the growth without PARPi. Data represents average and SEM for n=3 independent experiments. (e) Survival assay for the indicated breast (BC) or ovarian (OV) cancer cell lines after exposure for 24 h to taxol at the indicated concentrations. After removal of the drugs, cells were let grow for a week before harvest and counting. Quantifications of growth is normalized over the untreated conditions. Data from n=3 independent experiments, with mean and SD. (f-h) Quantification of survival of HCC-1937 cells (f), uWB1.289 cells expressing exogenous BRCA1 (g) or the original BRCA1-deficient uWB1.289 cells (h) after exposure to Taxol and PARPi at the indicated concentrations for 24 hrours. For HCC-1937 cells, PARPi was re-added for other 48 hours. After removal of the drugs, cells were let grow for a week before harvest. Quantifications of growth is normalized over the growth without PARPi. Data represents average and SEM for n=3 independent experiments. Statistical analysis by unpaired t-test: (**) $p<0.01$, (*) $p<0.05$, (ns) not significant.

Source data are provided as a Source Data file.
