# Supplementary material for: Nuclear deformability increases PARPi sensitivity in BRCA1-deficient cells by increasing microtubule-dependent DNA break mobility
Source: Nat Commun. 2025 Jun 17;16:5326. doi: 10.1038/s41467-025-60756-8 (PMC12174318; doi:10.1038/s41467-025-60756-8)
Supplement: Supplementary file 2 — Reporting Summary [file 41467_2025_60756_MOESM2_ESM.pdf]

nature portfolio

Corresponding author(s): Francisca Lottersberger

Last updated by author(s): 29/04/2025

Reporting Summary

Nature Portfolio wishes to improve the reproducibility of the work that we publish. This form provides structure for consistency and transparency in reporting. For further information on Nature Portfolio policies, see our Editorial Policies and the Editorial Policy Checklist.

Statistics

For all statistical analyses, confirm that the following items are present in the figure legend, table legend, main text, or Methods section.

n/a	Confirmed	
☐	☒	The exact sample size (*n*) for each experimental group/condition, given as a discrete number and unit of measurement
☐	☒	A statement on whether measurements were taken from distinct samples or whether the same sample was measured repeatedly
☐	☒	The statistical test(s) used AND whether they are one- or two-sided<br>*Only common tests should be described solely by name; describe more complex techniques in the Methods section.*
☒	☐	A description of all covariates tested
☐	☒	A description of any assumptions or corrections, such as tests of normality and adjustment for multiple comparisons
☐	☒	A full description of the statistical parameters including central tendency (e.g. means) or other basic estimates (e.g. regression coefficient) AND variation (e.g. standard deviation) or associated estimates of uncertainty (e.g. confidence intervals)
☐	☒	For null hypothesis testing, the test statistic (e.g. *F*, *t*, *r*) with confidence intervals, effect sizes, degrees of freedom and *P* value noted<br>*Give P values as exact values whenever suitable.*
☒	☐	For Bayesian analysis, information on the choice of priors and Markov chain Monte Carlo settings
☒	☐	For hierarchical and complex designs, identification of the appropriate level for tests and full reporting of outcomes
☒	☐	Estimates of effect sizes (e.g. Cohen's *d*, Pearson's *r*), indicating how they were calculated

Our web collection on statistics for biologists contains articles on many of the points above.

Software and code

Policy information about availability of computer code

Data collection	ChemiDoc (Bio-Rad), DeltaVision RT microscope system (GE Healthcare), Transmission electron microscope JEOL JEM1400 Flash (JEOL Ltd.)
Data analysis	GraphPad Prism (v9 or 10.0.0), Fiji (1.0), ImageJ 1.50i (Java 1.8.0) and plugins (StackReg, Mosaic Particle Tracker 2D/3D)

For manuscripts utilizing custom algorithms or software that are central to the research but not yet described in published literature, software must be made available to editors and reviewers. We strongly encourage code deposition in a community repository (e.g. GitHub). See the Nature Portfolio guidelines for submitting code & software for further information.

Data

Policy information about availability of data

All manuscripts must include a data availability statement. This statement should provide the following information, where applicable:

- Accession codes, unique identifiers, or web links for publicly available datasets
- A description of any restrictions on data availability
- For clinical datasets or third party data, please ensure that the statement adheres to our policy

All data generated or analyzed during this study are included in this article. Source data are provided with this paper. All raw images have been deposited in the Figshare database under accession code ZZ [add hyperlink here].

Research involving human participants, their data, or biological material

Policy information about studies with human participants or human data. See also policy information about sex, gender (identity/presentation), and sexual orientation and race, ethnicity and racism.

Reporting on sex and gender	N/A
Reporting on race, ethnicity, or other socially relevant groupings	N/A
Population characteristics	N/A
Recruitment	N/A
Ethics oversight	N/A

Note that full information on the approval of the study protocol must also be provided in the manuscript.

Field-specific reporting

Please select the one below that is the best fit for your research. If you are not sure, read the appropriate sections before making your selection.

☒ Life sciences ☐ Behavioural & social sciences ☐ Ecological, evolutionary & environmental sciences

For a reference copy of the document with all sections, see nature.com/documents/nr-reporting-summary-flat.pdf

Life sciences study design

All studies must disclose on these points even when the disclosure is negative.

Sample size	No statistical method was used to predetermine sample size. Sample size was determined based on previous similar experiments: n=30 metaphases over 3 independent experiments for mis-rejoined chromosomes (Lottersberger, F., Karssemeijer, R. A., Dimitrova, N. & de Lange, T. 53BP1 and the LINC Complex Promote Microtubule-Dependent DSB Mobility and DNA Repair. Cell 163, 880–893 (2015)), at least 20 nuclei over 3 independent experiments for mCherry-BP1 -2 foci mobility analysis (Lottersberger, F., Karssemeijer, R. A., Dimitrova, N. & de Lange, T. 53BP1 and the LINC Complex Promote Microtubule-Dependent DSB Mobility and DNA Repair. Cell 163, 880–893 (2015)), n=150 cells over 3 independent experiments for nuclei deformation analysis.
Data exclusions	No data were excluded from the experiments in the study but nuclei or metaphases where it was not possible to perform robust scoring (i.e.not clear/complete staining, less than 10 chromosomes, too many overlapping chromosomes) or growths where it was not possible to count colonies. In case of failed experiment (Cre or shRNA not working properly), all data associated with that specific experiment were not included.
Replication	The number of independent experiments performed is indicated in the figure legends. All sample images are representative of at least n=3 independent experiments with similar results, unless indicated.
Randomization	Randomization was done when possible for all the biological perturbation (Cre, shRNA, PARPi, Myriocin) by randomly choosing the culture dishes seeded at the same time with identical population. Pictures of metaphases and nuclei for all the cells/treatments were taken randomly by manual scanning of the slides/coverslips.For the other experiments, no randomization was applied. Appropiate controls were included where applicable.
Blinding	Investigators were not blinded during the study. However, all samples were processed in parallel and treated identically for all the experiments. In most experiments blinded analysis is not applicable since the sample identity is readily apparent to the investigator.

Reporting for specific materials, systems and methods

We require information from authors about some types of materials, experimental systems and methods used in many studies. Here, indicate whether each material, system or method listed is relevant to your study. If you are not sure if a list item applies to your research, read the appropriate section before selecting a response.

Materials & experimental systems

n/a	Involved in the study
☐	☒ Antibodies
☐	☒ Eukaryotic cell lines
☒	☐ Palaeontology and archaeology
☐	☒ Animals and other organisms
☒	☐ Clinical data
☒	☐ Dual use research of concern
☒	☐ Plants

Methods

n/a	Involved in the study
☒	☐ ChIP-seq
☒	☐ Flow cytometry
☒	☐ MRI-based neuroimaging

Antibodies

Antibodies used	immunoblots:<br>Mouse monoclonal beta-Actin (#3700; Cell Signal, 1:2000)<br>Mouse monoclonal Chk2 (BD 611570; BD Biosciences, 1:200)<br>Mouse monoclonal h/mBRCA1 (MAB22101, R&D system 1:200)<br>Rabbit monoclonal 53BP1 (ab175933, Abcam, 1:1000)<br>Rabbit polyclonal SPT1 (ab176706, Abcam, 1:750)<br>Mouse monoclonal Lamin B1 (sc374015, Santacruz, 1:750)<br>Mouse monoclonal Lamin A/C (4777, Cell Signaling, 1:1000)<br>Mouse monoclonal p53 (ab90363, Abcam, 1:1000)<br>Donkey polyclonal anti-Rabbit igG HRP (Cytiva, NA934V; 1:1000-1:2000)<br>Goat polyclonal anti-rabbit IgG HRP (Invitrogen 31460, 1:1000-2000)<br>Goat polyclonal anti-mouse IgG HRP (Invitrogen 31430, 1:1000-2000)<br>Immunofluorescence:<br>Mouse monoclonal Lamin B1 (sc374015, Santacruz, 1:500)<br>Mouse monoclonal Lamin A/C (sc518013, Santacruz 1:250)<br>Mouse monoclonal Lamin A/C (4777, Cell Signaling, 1:1000)<br>Donkey polyclonal anti-Mouse igG, AlexaFluorTM 488/647 (A21202/A-21235, Invitrogen, 1:1000),
Validation	All the antibodies used were validated by the suppliers companies for reactivity against the mouse or human proteins by Immunoblot or Immunofluorescence.<br>Antibodies against h/mBRCA1 (MAB22101, R&D system), SPT1 (ab176706, Abcam), Lamin B1 (sc374015, Santacruz), Lamin A/C (4777, Cell Signaling, and sc518013, Santacruz and p53 (ab90363, Abcam) were confirmed in this study by western blot of Crispr/Cre-mediated knockout, shRNA-mediated knock down, Transnetyx Inc-confirmed knock out, confirming the loss of the relevant bands.

Eukaryotic cell lines

Policy information about cell lines and Sex and Gender in Research

Cell line source(s)	293T/17 [HEK 293T/17] (CRL-11268) and Phoenix ECO cells (CRL-3214) were obtained by ATCC, Rockville, MD). MCF7, MDA-MB-231 were a gift from Charlotta Dabrosin. UWB1.289 (ATCC CRL-2945), UWB1.289+BRCA1 (ATCC CRL-2946) and HCC1937 (DSMZ ACC 513) were obtained from IFOM Human biorepository unit.<br>MEFs used were generated for this study.
Authentication	No authentication was performed for 293T and Phoenix ECO cells.<br>MEFs were genotyped by Transnetyx Inc. using real-time PCR and authenticated by Immunoblots for LMNA/C and BRCA1 deletion. Human cancer cell lines were authenticated when possible by Immunoblots (for BRCA1 levels). Sex was not assessed.
Mycoplasma contamination	All cells tested negative for Mycoplasma contamination
Commonly misidentified lines<br>(See ICLAC register)	No commonly misidentified cell lines were used in this study.

Animals and other research organisms

Policy information about studies involving animals; ARRIVE guidelines recommended for reporting animal research, and Sex and Gender in Research

Laboratory animals	Pregnant female mice were used to isolate MEFs from E12.5 embryos.<br>Species Mus musculus musculus;; Strain mixed C57BL/6 and 129 ; sex female and male; age range 2-10 months.<br>Lmna+/- mice (009125, the Jackson Lab) and Brca1F/F (Xu et al., Mol Cell, 1999) mice were used to derive all the genotypes by standard crosses.
Wild animals	N/A

Reporting on sex	N/A
Field-collected samples	N/A
Ethics oversight	Mice were housed and cared for under the Rockefeller University AIACUC protocol 16865-H at the Rockefeller University's Comparative Bioscience Center, which provides animal care according to NIH guidelines.

Note that full information on the approval of the study protocol must also be provided in the manuscript.
